# Drift and Hysteresis Characteristics of Drug Sensors Based on Ruthenium Dioxide Membrane

**DOI:** 10.3390/s8095386

**Published:** 2008-09-03

**Authors:** Yi-Hung Liao, Jung-Chuan Chou

**Affiliations:** 1 Graduate School of Engineering Science and Technology, National Yunlin University of Science and Technology / 123, 3 sec. University Rd., Douliou, Yunlin, Taiwan; 2 Department of Information Management, Transworld of Institute of Technology / 1221, Jen-Nang Rd., Chia-Tong Li, Douliou, Yunlin, Taiwan; E-mail: liaoih@tit.edu.tw; 3 Graduate School of Electronic Engineering, National Yunlin University of Science and Technology/ 123, 3 sec. University Rd., Douliou, Yunlin, Taiwan; E-mail: choujc@yuntech.edu.tw

**Keywords:** Procaine drug sensor, berberine drug sensor, ruthenium dioxide, drift rate, hysteresis width

## Abstract

The drug sensing properties of procaine and berberine drug sensors based on ruthenium dioxide thin film were investigated. Ruthenium dioxide (RuO_2_) membrane prepared using a sputtering method was used as substrates for the drug sensors. The procaine and berberine drug sensors were prepared using a drug-saensitive membrane that measured the procaine and berberine concentration in a linear range from 1×10^-2^ M to 1×10^-6^ M and from 1×10^-2^ M to 1×10^-7^ M, respectively. The drift rates and hyteresis widths of these ruthenium dioxide based drug sensors were also investigated.

## 1. Introduction

In this decade, many researchers have used metal-oxide materials for pH sensors [1-4] or biosensors [5-8]. Ruthenium dioxide (RuO_2_) is a material of great interest because it has some advantages such as high conductivity, electrochemical reversibility, good of enzyme and compound adhesion and is very stable in acidic solvents. We used the ruthenium dioxide thin film as the substrate for procaine and berberine drug sensors. Procaine, molecular formula C_13_H_20_N_2_O_2_, is used chiefly in its hydrochloride form as a local anesthetic in medicine and dentistry. It was first synthesized in 1905 by the German chemist Albert Einhorn. He was looking for a compound that would act as an anesthetic, be nontoxic, free of side effects, and not be addictive. Dozens of works [9-12] have investigated procaine drug sensors using different substrates. The electrochemical behavior of procaine at the multi-walled nanotube (MWNT) film coated glassy carbon electrode was investigated [9]. Bergamini *et al.* [10] reported that procaine determination was possible with flow injection analysis (FIA) using a screen-printed carbon electrode. Chou *et al.* [11] presented the procaine characteristics of the extended gate field effect transistor based on indium tin oxide (ITO) glass. An ion-selective sensor for procaine hydrochloride based on a piezoelectric quartz crystal was studied [12]. Berberine is a plant alkaloid with a long history of medicinal use in both Ayurvedic and Chinese medicine. It is present in a number of clinically important medicinal plants. Berberine has been shown to exhibit significant antimicrobial activity against a variety of bacteria [13]. Among Chinese herbs, the primary sources are phellodendron and coptis. Yang *et al.* [14] reported on an optical sensor used for determining berberine. Another berberine sensitive optical fiber sensor was prepared using a composite active material investigated by Zhang *et al.*[15]. Yang *et al.* [16] presented a cetyltrimethylammonium assay using a berberine fluorescence probe. Huang *et al.* [17] reported a sensitive optode membrane for berberine using a conjugated polymer. Liu *et al.* [18] also adopted an optical fiber sensor based on immobilized 1, 4-bis (naphtha[2,1-d]oxazole-2-yl)benzene for berberine. Hsiung *et al.* [19] presented an extended gate field effect transistor based on SnO_2_/ITO to analyze berebrine. In this study, the ruthenium dioxide thin film was deposited using a sputtering system and used for procaine and berberine drug sensors. Furthermore, the sensing and nonideal (drift rate and hysteresis width) characteristics of these drug sensors were investigated.

## 2. Experimental

### 2.1. Materials and regents

The ruthenium dioxide thin film substrate is a silicon wafer. The silicon substrate was cleaned using ethanol and acetone solvents in an ultrasonic unit. The ethanol and acetone regents were purchased from Katayama (Japan). Procaine hydrochloride (C_13_H_20_O_2_N_2_·HCl, FW: 272.77, powder) was used for preparing procaine solutions and was obtained from ACROS (USA). Berberine chloride was used to prepare berberine solutions and was purchased from Fluka (Switzerland). Some chemical materials used for preparing the sensing membrane consist of polyvinyl chloride polymer (PVC) ((-CH_2_:CHCl-)_n_, powder) obtained from Showa (Japan). The di-octyl-phthalate (DOP) (C_24_H_38_O_4_, M = 390.56, solution) was purchased from Shimakyu's Pure Chemicals (Osaka, Japan), phosphotungstic acid (H_3_PW_12_O_40_·H_2_O, M = 2880.17, powder) was purchased from Aldrich Chemical Co. Ltd (USA). Tetrahydrofuran (THF) (C_4_H_8_O, FW: 72.11,) was obtained from Katayama (Japan).

### 2.2. Fabrication of ruthenium dioxide membrane

**Figure 1.**
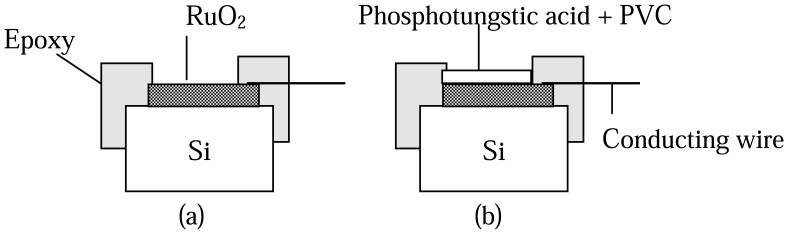
(a) Cross section of ruthenium dioxide membrane. (b) Cross section of procaine and berberine drug sensors.

The ruthenium dioxide thin film was deposited onto a silicon substrate using a sputtering system. The silicon wafers were cleaned ultrasonically in acetone and ethanol alternately for 15 minutes, leached in distilled (D.I.) water and then dried. The total operating pressure of the sputtering system was 10 mtorr in Ar-gas-mixed O_2_ for 15 minutes. The gas flow ratio of the Ar: O_2_ was 40:22 in sccm. The radio frequency power was 100W, at 13.56MHz. After the sputtering was completed, the thin film was removed from the chamber. Wafers were cut into 0.5 cm × 0.5 cm size chips and packaged with epoxy resin. A cross section of the ruthenium dioxide ion selective electrode is shown in Fig. 1(a).

### 2.3. Preparation of drug sensors

The drug sensors were prepared using a ruthenium dioxide ion selective electrode. The drug-sensitive membrane was prepared using a mixture solution, which included polyvinyl chloride polymer, phosphotungstic acid, diocyl phthalate plasticizer and tetrahydrofuran solvent. The weight ratios of the PVC, phosphotungstic acid and diotcyl phthalate plasticizer were 4:2.5:5, respectively. The procaine and berberine react with the phosphotungstic acid in solution. About 1 microliter (μL) of the mixture solution was dropped onto the sensitive area (2 mm × 2 mm) and allowed to dry at room temperature for about 12 hours. When the sensitive membrane was dried, the drug sensor devices were placed into 10^-2^ M of procaine and berberine solution for about 24 hours, respectively. The sensing characteristics of the procaine and berberine drug sensors were carried out using the procaine and berberine concentrations, respectively. A cross section of the procaine and berberine drug sensors is shown in Fig. 1(b).

### 2.4. Measurement system

**Figure 2.**
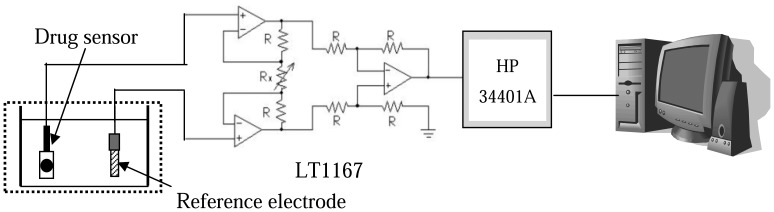
Voltage-time measurement system used for drug sensors

Figure 2 shows the measurement response voltage versus time for procaine and berberine drug sensors. The drug sensors and reference electrode were immersed in procaine and berberine solutions. A drug sensor measurement system consists of an instrument amplifier readout circuit (LT1167) and a digital multifunction meter (HP 34401A). The gain of the instrument amplifier is equal to 1. In this study, the drug sensor sensitivity and drift, hysteresis effects were investigated using a V-T measurement system. The drug sensors and Ag/AgCl reference electrode were immersed in procaine concentration from 10^-2^ M to 10^-6^ M and berberine concentration from 10^-2^ M to 10^-7^ M, respectively. And we recorded the response voltage with different concentrations to obtain the sensitivity of drug sensors. In the drift test, the drug sensors and Ag/AgCl reference electrode were immersed in the 10^-2^ M concentration for 12 hours. Then we used the digital meter to record the output voltage of the drug sensors. After 5 hours, the response of drug sensors is completed. Therefore, drift rate is the slope of output voltage with respect to time, where the time is greater than 5 hours. Use the voltage-time measurement system to measure the hysteresis width of drug sensors in different concentrations with a loop cycle.

## 3. Results and Discussion

### 3.1. Sensitivities of the drug sensors

In this study, we utilized a ruthenium dioxide thin film as a drug sensitive membrane substrate. The drug sensitive membrane was prepared by using the phosphotungstic acid and polyvinyl chloride (PVC) to immobilize onto the ruthenium dioxide thin film substrate and form the corresponding procaine and berberine drug sensors. The procaine and berberine solutions were prepared with procaine hydrochloride, berberine chloride and deionized (D.I.) water. The procaine hydrochloride and berberine chloride are alkaloids and the phosphotungstic acid is an electroactive material. Alkaloids are presented as *RNH_2_* and the electric active material is shown as *A.HX*. The reactions on anion exchangers are explained either by the absorption of acid or by anion exchange. Most probably both reactions are taking place to a certain extent. When determining alkaloid salts on the anion exchanger the following exchange may be considered [20]:

(1) $$\left(RNH_{2}/H_{3}O+OH^{-}\right)+A.HX+H_{2}O\overset{K}{↔}\left(RNH_{2}/H_{3}O/X^{-}\right)+A+2H_{2}O$$

After the reaction, we can obtain the alkaloid concentration by the amount of salt precipitation. The polyvinyl chloride is a polymeric material and is used to entrap electroactive sensing materials. Phosphotungstic acid is such an electroactive material and can be reacted with procaine hydrochloride and berberine chloride. Therefore, the drug sensitive membrane was entrapped phosphotungstic acid using polyvinyl chloride to determine the procaine and berebrine solutions.

**Figure 3.**
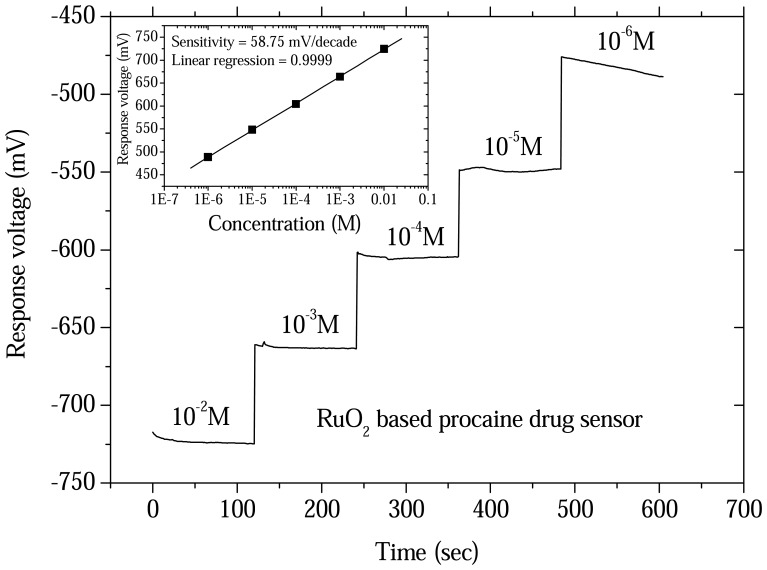
Response voltage versus time characteristics and sensitivity of ruthenium dioxide based procaine drug sensor from 10^-2^ M to 10^-6^ M concentrations

**Figure 4.**
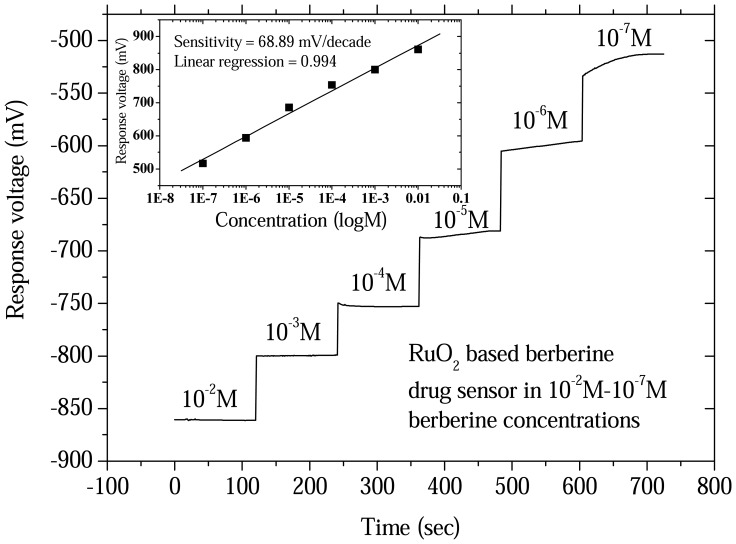
Response voltage versus time characteristics and sensitivity of ruthenium dioxide based berberine drug sensor from 10^-2^ M to 10^-7^ M concentrations.

The sensitivities of the procaine and berberine drug sensors were obtained from the response voltage versus time with a V-T measurement system. The drug sensors and reference electrode were immersed in different concentrations of procaine and berberine, respectively. The measurement data used Origin 6.1 software to obtain a response versus time plot. The response voltages versus time characteristics for the procaine drug sensor from 10^-2^ M to 10^-6^ M concentrations are shown in Fig. 3. The response voltages versus time characteristics of berberine drug sensor from10^-2^ M to 10^-7^ M concentrations are shown in Fig. 4. The procaine drug sensor had a measured linear range from 10^-2^ M to 10^-6^ M with a detection limit at 2×10^-7^ M. The sensitivity of the procaine drug sensor was derived at 58.75mV/decade with good linearity. The experimental results are shown in the inset of Fig. 3. The linear measurement range for the berberine drug sensors were from 10^-2^ M to 10^-7^ M with the detection limit at 2×10^-8^ M. The sensitivity of the berberine drug sensor was obtained at 68.89mV/decade. The experimental result is shown in the inset of Fig. 4. We also compared detection range (limit) of the procaine and berberine drug sensors with the previous studies [9-12, 14-19] and the results are shown in Table 1.

### 3.2. Drift rates of drug sensors

The output response of drug sensors reached stability in the measurement process. However, the output voltage of sensor will slowly change proportionally with time during long term monitoring and this problem still remains unsolved. This phenomenon is named drift effect. For a long term measurement of the drug sensors, the drift behaviors of drug sensors can be described as multiple time-constant models as follows [21-23]:

(2) O(t)=0,fort<0

(3) $$O\left(t\right)=S_{pC}\Delta pC\left[1-\sum_{i=1}^{n}\varepsilon_{i}\exp\left(-\frac{t}{\tau_{i}}\right)\right],\;\text{for}\;t>0$$

where *O*(*t*) is the slow response, S_pC_ is the total pC sensitivity, ΔpC is the value of the pC step, and ε*_i_* and τ*_i_* are the normalized amplitude and time constant of the corresponding exponential term *i*, respectively. The term ε[1-exp(-t/τ)] presents the slow response.

**Figure 5.**
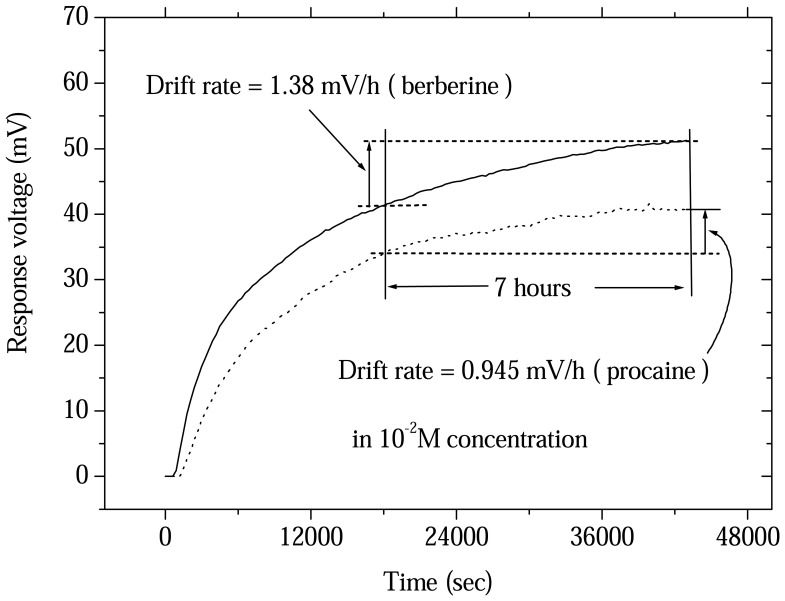
Drift rates of procaine and berberine drug sensors based on ruthenium dioxide membrane in 10^-2^ M concentration measurement between 5 and 12 hours.

Nonideal effects existed in different sensors. The drift rate for the sensor is very important parameter in the measurement. In the long-term measurement process, after a drug sensor is stable. The sensor response voltage slowly varies with time. In this research, according to previous paper described [24] the drug sensor was stable after 5 hours. The drift rate of the drug sensor was obtained from the difference in response voltage between 7 and 12 hours. In the experimental results were shown in Fig. 5, the drift rate of procaine drug sensor was 0.945 mV/h in 10^-2^ M procaine concentration. The drift rate of berberine drug sensor was 1.38 mV/h in 10^-2^ M berberine concentration.

### 3.3. Hysteresis width of drug sensors

As test solution concentration change after resumption originally buffer solution, at twice measuring will exist a voltage difference, which is named hysteresis width. Based on slow response mechanism of drug sensors, a hysteresis was derived when the pC is ramped up and down [25]. The normalized maximum width of the hysteresis loop was described as follow [25, 26]

(4) $$W_{\max}=2\varepsilon r\left\{1-\frac{2\exp\left(0.5/r\right)}{1+\exp\left(1/r\right)}\right\}$$

where *r* is the determinative parameter defined as *r* = τ/t_s_, where t_s_ is the time needed for a sweep from one pC extreme to another.

**Figure 6.**
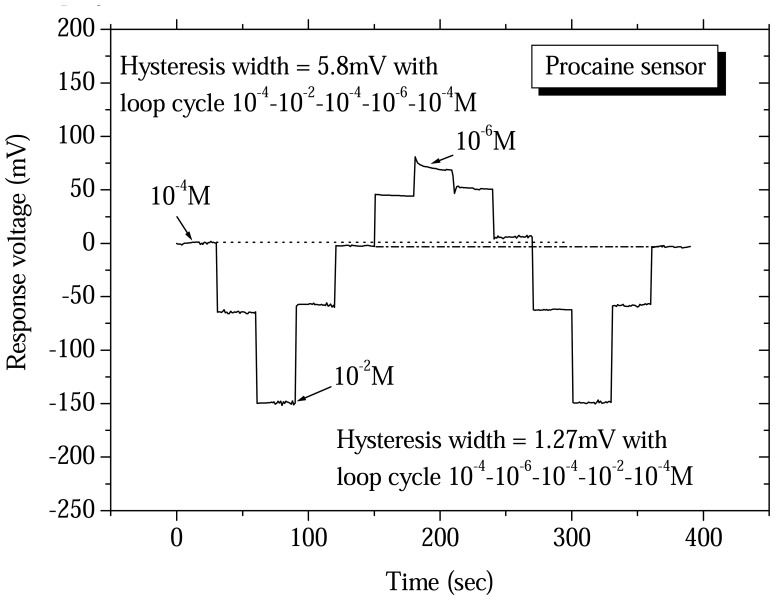
Hysteresis widths of procaine drug sensors based on ruthenium dioxide membrane with the 10^-4^-10^-2^-10^-4^-10^-6^-10^-4^ M and the 10^-4^-10^-6^-10^-4^-10^-2^-10^-4^ M of procaine concentrations loop cycle.

**Figure 7.**
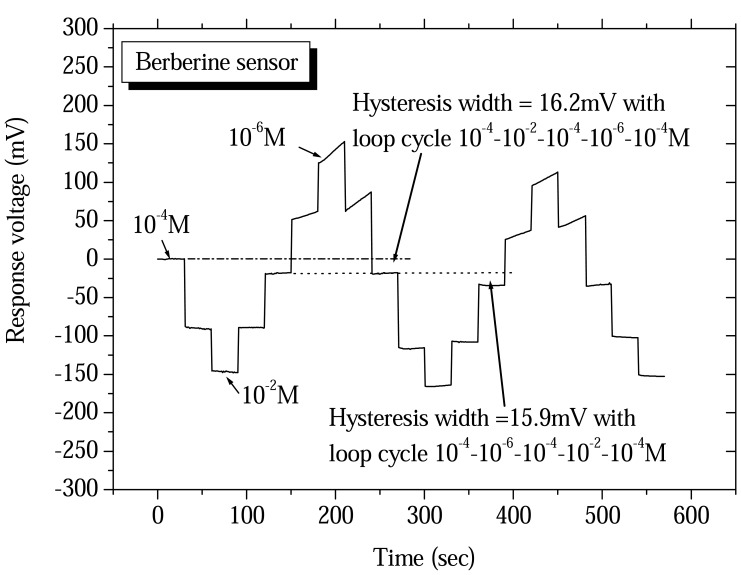
Hysteresis widths of berberine drug sensors based on ruthenium dioxide membrane with the 10^-4^-10^-2^-10^-4^-10^-6^-10^-4^ M and the 10^-4^-10^-6^-10^-4^-10^-2^-10^-4^ M of berberine concentrations loop cycle.

The hysteresis effect was another non-ideal effect in the drug sensor in repeated measurements. According to Bousse *et al.*[27] the hysteresis of pH-ISFET could be regarded as a delay of the pH response. Used this principle, the hysteresis of drug sensor also could be regarded as a delay of the concentration response. The hysteresis measurement widths for the procaine and berberine drug sensors were determined using a V-T measurement system. The drug sensor and Ag/AgCl reference electrode were placed in different aqueous solution concentrations. We selected the loop cycle for measurement to obtain hysteresis widths of the procaine and berberine drug sensors and the results are shown in Figures 6 and 7. According to the experimental results, the hysteresis widths of procaine drug sensor were acquired at 5.8mV and 1.27mV with the 10^-4^-10^-2^-10^-4^-10^-6^-10^-4^ M and the 10^-4^-10^-6^-10^-4^-10^-2^-10^-4^ M of procaine concentrations loop cycle, respectively. The hysteresis widths of berberine drug sensor were obtained at 16.2mV and 15.9mV with the 10^-4^-10^-2^-10^-4^-10^-6^-10^-4^ M and the 10^-4^-10^-6^-10^-4^-10^-2^-10^-4^ M of berberine concentrations loop cycle, respectively.

## 4. Conclusions

Ruthenium dioxide sensing membrane was prepared using a sputtering system. Fabrication and measurement of procaine and berberine drug sensors using ruthenium dioxide ion selective electrodes were investigated. We obtained the sensitivities of procaine and berberine drug sensors at 58.75 mV/decade and 68.89 mV/decade, respectively. The drift and hysteresis of the nonideal characteristics of procaine and berebrine drug sensors were also studied. The drift rate of the procaine sensor was 0.945 mV/h in 10^-2^ M procaine concentration. The drift rate of the berberine sensor was 1.38 mV/h in 10^-2^ M berberine concentration. The hysteresis widths of procaine drug sensor were acquired at 5.8 mV and 1.27mV with the 10^-4^-10^-2^-10^-4^-10^-6^-10^-4^ M and the 10^-4^-10^-6^-10^-4^-10^-2^-10^-4^ M of procaine concentrations loop cycle, respectively. The hysteresis widths of berberine drug sensor were obtained at 16.2 mV and 15.9 mV with the 10^-4^-10^-2^-10^-4^-10^-6^-10^-4^ M and the 10^-4^-10^-6^-10^-4^-10^-2^-10^-4^ M of berberine concentrations loop cycle, respectively.

## Figures and Tables

**Table 1.** Comparison of linear detection range and detection limit of procaine and berberine drug sensors.

Procaine			

substrate	Linear detection range	Detection limit	Ref.

RuO_2_/Si	1×10^-6^ M-1×10^-2^ M	2×10^-7^ M	In this study
MWNT/GCE	5×10^-7^ M-1×10^-4^ M	2×10^-7^ M	[9]
Carbon electrode	9×10^-6^ M-1×10^-4^ M	6×10^-6^ M	[10]
Indium tin oxide	1×10^-6^ M-1×10^-2^ M	-	[11]
Piezoelectric quartz crystal	8.3×10^-8^ M-5.0×10^-3^ M	8.3×10^-8^ M	[12]
Berberine			

substrate	Linear detection range	Detection limit	Ref.

RuO_2_/Si	1×10^-7^ M - 1×10^-2^ M	2×10^-8^ M	In this study
Quartz plate	4×10^-7^ M - 2×10^-5^ M	8×10^-8^ M	[14]
Quartz plate	7.5×10^-7^ M - 5.6×10^-4^ M	-	[15]
Quartz plate	7×10^-1^ M - 4×10^-9^ M	3×10^-11^ M	[16]
Conjugated polymer	7.5×10^-7^ M - 7.5×10^-4^ M	-	[17]
Copolymer	4.02×10^-7^ M - 2.82×10^-4^ M	-	[18]
SnO_2_/ITO	5×10^-7^ M - 1×10^-3^ M	-	[19]
